# Supplementary material for: Predictive coding networks for temporal prediction
Source: PLoS Comput Biol. 2024 Apr 1;20(4):e1011183. doi: 10.1371/journal.pcbi.1011183 (PMC11008833; doi:10.1371/journal.pcbi.1011183)
Supplement: S1 Appendix — (PDF) [file pcbi.1011183.s001.pdf]

Supporting Information for Predictive Coding Networks for Temporal Prediction

Beren Millidge[1◑], Mufeng Tang[1◑], Mahyar Osanlouy[2], Nicol S. Harper[3], Rafal Bogacz[1*]

1 MRC Brain Network Dynamics Unit, University of Oxford, Oxford, UK
2 Auckland Bioengineering Institute, University of Auckland, Auckland, New Zealand
3 Department of Physiology, Anatomy and Genetics, University of Oxford, Oxford, UK

◑These authors contributed equally to this work.
* rafal.bogacz@bndu.ox.ac.uk

S1 Appendix. Derivation of recursive Bayesian estimation. Here we provide detailed derivations of Equation 16, showing that the original Bayesian filtering problem can be simplified as a recursive estimation process that finally leads to Kalman filtering and our own temporal predictive coding:

$$\begin{aligned}
p(x_k|y_{1:k}) &= \int p(x_{1:k}|y_{1:k})dx_1,\ldots,dx_{k-1} \\
&\overset{(a)}{\propto} \int p(y_{1:k}|x_{1:k})p(x_{1:k})dx_1,\ldots,dx_{k-1} \\
&\overset{(b)}{=} \int \prod_{i=1}^{k} p(y_i|x_i)p(x_i|x_{i-1})dx_1,\ldots,dx_{k-1} \\
&= p(y_k|x_k)\int p(x_k|x_{k-1})\prod_{i=1}^{k-1} p(y_i|x_i)p(x_i|x_{i-1})dx_1,\ldots,dx_{k-1} \\
&= p(y_k|x_k)\int p(x_k|x_{k-1})\left[\int \prod_{i=1}^{k-1} p(y_i|x_i)p(x_i|x_{i-1})dx_1,\ldots,dx_{k-2}\right]dx_{k-1} \\
&\overset{(c)}{\propto} p(y_k|x_k)\int p(x_k|x_{k-1})p(x_{k-1}|y_{1:k-1})dx_{k-1}
\end{aligned} \tag{1}$$

where step (a) is achieved by Bayes' rule, step (b) is achieved by the Markov assumption we made on the hidden states, and step (c) is achieved by observing that the integral in the square bracket in the penultimate line is proportional to the Bayesian filtering posterior at the previous step $k-1$. This can be observed by comparing it with the expression on the third line of the above equations. Importantly, this expression provides a recursive solution to the problem i.e., knowing the posterior at step $k-1$, we could estimate the posterior at step k.
