# Supplementary material for: Predictive coding networks for temporal prediction
Source: PLoS Comput Biol. 2024 Apr 1;20(4):e1011183. doi: 10.1371/journal.pcbi.1011183 (PMC11008833; doi:10.1371/journal.pcbi.1011183)
Supplement: S2 Appendix — (PDF) [file pcbi.1011183.s002.pdf]

# Supporting Information for Predictive Coding Networks for Temporal Prediction

Beren Millidge[1☯], Mufeng Tang[1☯], Mahyar Osanlouy[2], Nicol S. Harper[3], Rafal Bogacz[1*]

**1** MRC Brain Network Dynamics Unit, University of Oxford, Oxford, UK
**2** Auckland Bioengineering Institute, University of Auckland, Auckland, New Zealand
**3** Department of Physiology, Anatomy and Genetics, University of Oxford, Oxford, UK

☯These authors contributed equally to this work.
* rafal.bogacz@bndu.ox.ac.uk

**S2 Appendix. Derivation of the update rules for the models.** Here we derive the update rule for $\hat{x}_k$ that underlies both predictive coding and Kalman filtering (Equations 22 and 26). Notice that the objective functions for these two models follow a unified form:

$$\mathcal{F}_k = \frac{1}{2}(y_k - Cx_k)^T \Sigma_y^{-1}(y_k - Cx_k) + \frac{1}{2}(x_k - \hat{x}_k^-)^T S^{-1}(x_k - \hat{x}_k^-) \tag{1}$$

where $S = \Sigma_x$ for predictive coding and $S = A\Sigma_{k-1}A^T + \Sigma_x$ for Kalman filtering. To obtain $\hat{x}_k$ that minimizes $\mathcal{F}_k$, we first take the derivative of $\mathcal{F}_k$ with respect to $x_k$:

$$\frac{\partial \mathcal{F}_k}{\partial x_k} = (C^T\Sigma_y^{-1}C + S^{-1})x_k - (C^T\Sigma_y^{-1}y_k + S^{-1}\hat{x}_k^-). \tag{2}$$

Then, by setting the derivative to 0 we have the optimal $\hat{x}_k$:

$$\begin{aligned}
\hat{x}_k &= (C^T\Sigma_y^{-1}C + S^{-1})^{-1}(C^T\Sigma_y^{-1}y_k + S^{-1}\hat{x}_k^-) \\
&\overset{(a)}{=} [S - SC^T(\Sigma_y + CSC^T)^{-1}CS](C^T\Sigma_y^{-1}y_k + S^{-1}\hat{x}_k^-) \\
&\overset{(b)}{=} [S - KCS](C^T\Sigma_y^{-1}y_k + S^{-1}\hat{x}_k^-) \\
&= \hat{x}_k^- - KC\hat{x}_k^- + [SC^T\Sigma_y^{-1} - KCSC^T\Sigma_y^{-1}]y_k \\
&= \hat{x}_k^- - KC\hat{x}_k^- + [KK^{-1}SC^T\Sigma_y^{-1} - KCSC^T\Sigma_y^{-1}]y_k \\
&= \hat{x}_k^- - KC\hat{x}_k^- + K[(\Sigma_y + CSC^T)C^{-T}S^{-1}SC^T\Sigma_y^{-1} - CSC^T\Sigma_y^{-1}]y_k \\
&= \hat{x}_k^- - KC\hat{x}_k^- + Ky_k \\
&= \hat{x}_k^- + SC^T(\Sigma_y + CSC^T)^{-1}(y_k - C\hat{x}_k^-).
\end{aligned} \tag{3}$$

In step (a) we use the Woodbury matrix inversion identity and in step (b) we replace $SC^T(\Sigma_y + CSC^T)^{-1}$ with $K$. Substituting $S = \Sigma_x$ for predictive coding and $S = A\Sigma_{k-1}A^T + \Sigma_x$ for Kalman filtering we get Equations 22 and 26 respectively.
