# Supplementary material for: Predictive coding networks for temporal prediction
Source: PLoS Comput Biol. 2024 Apr 1;20(4):e1011183. doi: 10.1371/journal.pcbi.1011183 (PMC11008833; doi:10.1371/journal.pcbi.1011183)
Supplement: S3 Appendix — (PDF) [file pcbi.1011183.s003.pdf]

Supporting Information for Predictive Coding Networks for Temporal Prediction

Beren Millidge[1◑], Mufeng Tang[1◑], Mahyar Osanlouy[2], Nicol S. Harper[3], Rafal Bogacz[1*]

1 MRC Brain Network Dynamics Unit, University of Oxford, Oxford, UK
2 Auckland Bioengineering Institute, University of Auckland, Auckland, New Zealand
3 Department of Physiology, Anatomy and Genetics, University of Oxford, Oxford, UK

◑These authors contributed equally to this work.
* rafal.bogacz@bndu.ox.ac.uk

S3 Appendix. Experiments on a simple nonlinear model. In this Appendix, we focus on a simple simulation in which observations are generated from a probabilistic model of the same form as that of a nonlinear predictive coding network. In such a setting we expected the nonlinear temporal predictive coding network to learn to predict these data well and we examined whether it is able to do it better than a linear model.

We generated a time series of 100, two-dimensional stimuli according to Eqs. 1 and 2 in the main text where the nonlinear function f is chosen to be the hyperbolic tangent function, and ω_y and ω_x correspond to standard Gaussian noise sampled from an i.i.d. Gaussian distribution with a mean of 0 and a variance of 0.01. In this 2D example, parameters of C and A were set to a rotation matrix and the identity matrix multiplied by 3, respectively, which are as follows,

$$\begin{aligned} A &= \begin{bmatrix} \frac{-1}{2}\Delta k & 1 \\ -1 & \frac{-1}{2}\Delta k \end{bmatrix} \times 3 \\ C &= \begin{bmatrix} 1 & 0 \\ 0 & 1 \end{bmatrix} \times 3. \end{aligned} \tag{1}$$

The multiplication by 3 in both matrices above was performed to produce a system that operates beyond the linear range of the hyperbolic tangent function (we used $\Delta k = 0.5$). The observed stimuli are periodic signals which are simply noisy versions of the hidden variables.

Both linear and nonlinear models were trained to predict these stimuli. The models were simulated with a single state of inference between samples ($\Delta k = \Delta t$). For the learning process, the parameters of the C matrix were initialized as an identity matrix while the weights between hidden units were initialized to zeros. Additionally, each neuron $\hat{x}$ was initialized to 0, and the model was trained according to Equations 11 in the main text.

Fig S3A and B show zoomed-in time series with predictions of nonlinear and linear models (for the sake of visualization, only one dimension is shown). Fig S3A demonstrates that in the first 50 time steps of the simulation, both models significantly move their predictions towards true time-series. Fig S3B shows the final 50 time steps, where the nonlinear model performed relatively better than the linear model.

The observation prediction error of each model is depicted in Fig S3C. Within the first 50 to 100 time steps, the linear model has a much higher rate of reduction in its prediction error compared with the nonlinear model; however, this rapid drop in the error is followed by a stationary line and even a slow divergence from the optimum toward the end of the simulation. On the other hand, the nonlinear model shows a

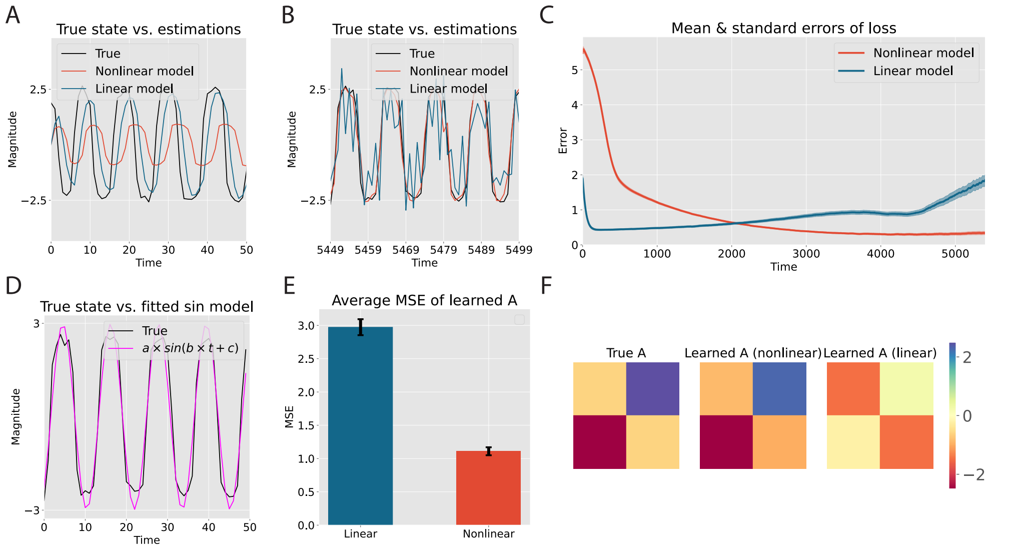

S3 Figure. Results with the simple nonlinear model. A, B: Predictions in the first and final 50 steps of the models. C: MSE of both models as a function of time. D: Fitted values with the sin function. E, F: MSE between the learnt As and the true A and visualisations of the learnt and true As.

slower, but relatively steady, rate of error reduction which ultimately leads to convergence.

The lower loss achieved by the nonlinear model could be explained by the fact that the ground-truth simulation data has a different oscillatory pattern as a result of the nonlinearity in the Eqs 1 and 2 that were used to generate such data. Therefore, the shape of the signal is different than one would expect to observe from a simple linear system. To show this, we simulated a data generation process based on the following equation:

$$\hat{y}_k = a \sin(bk + c), \tag{2}$$

where a, b, and c were approximated by a least squares optimisation so they match the ground-truth data. The comparison is depicted in Figure S3D, illustrating the difference in the shape of the time-series.

To better understand the behaviour of each model, we investigated how well the transition matrix A is learnt in each case. In Fig S3F, we plotted the mean of the learnt matrix by colour-coding the elements and normalising it to the minimum and maximum values of the true matrix. As expected, the parameters of the A matrix are closer to the true values when compared against their corresponding values in the linear model. To quantify it, we also plotted the average errors of the learnt parameters for both models, showing that the linear model has a much higher error (almost by three folds) versus the nonlinear model - see Fig S3E.
