# Supplementary figures and images for: Predictive coding networks for temporal prediction
Source: PLoS Comput Biol. 2024 Apr 1;20(4):e1011183. doi: 10.1371/journal.pcbi.1011183 (PMC11008833; doi:10.1371/journal.pcbi.1011183)

# Estimated Position

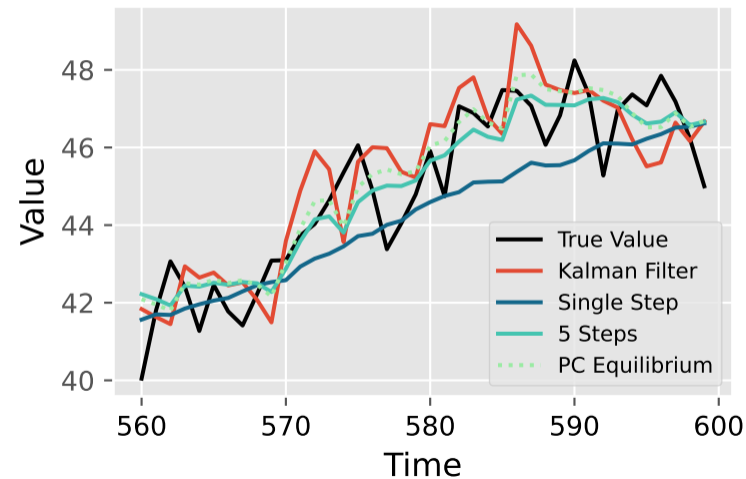

# Estimated Velocity

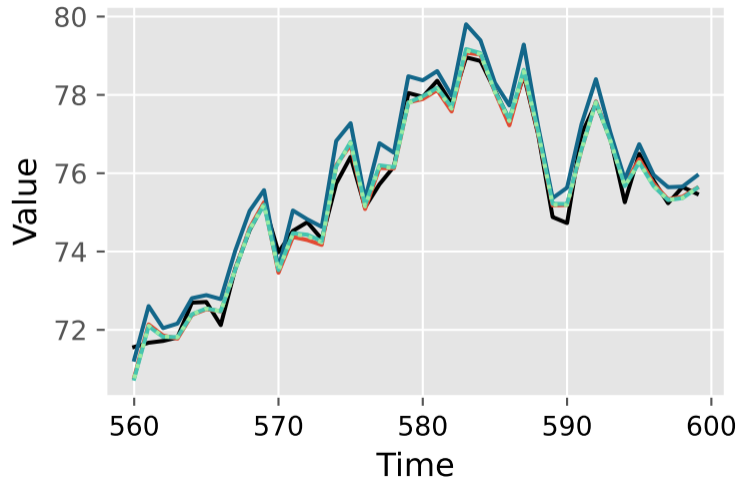

Supplement: S1 Fig — All values are with arbitrary units (a.u.). (PDF) [file pcbi.1011183.s004.pdf]

State

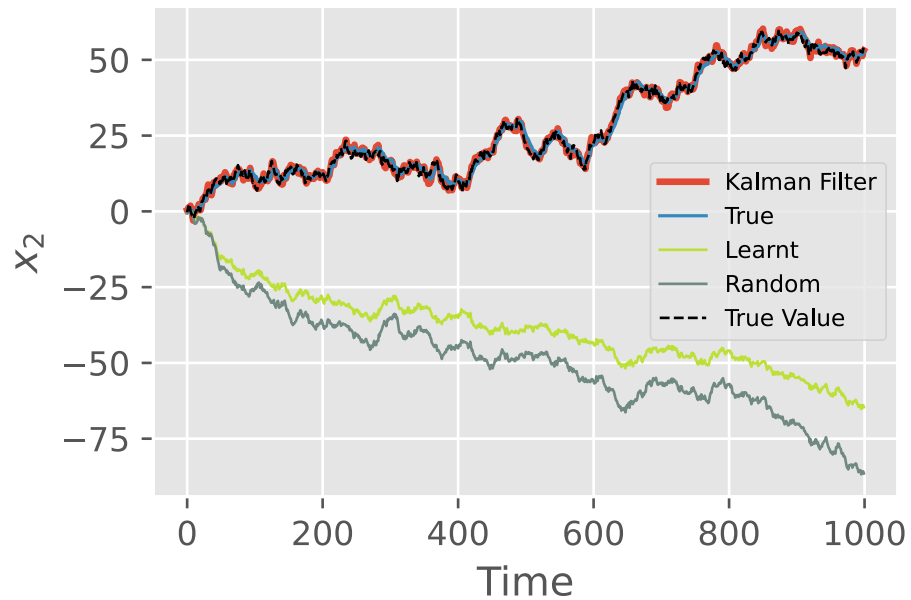

Observed

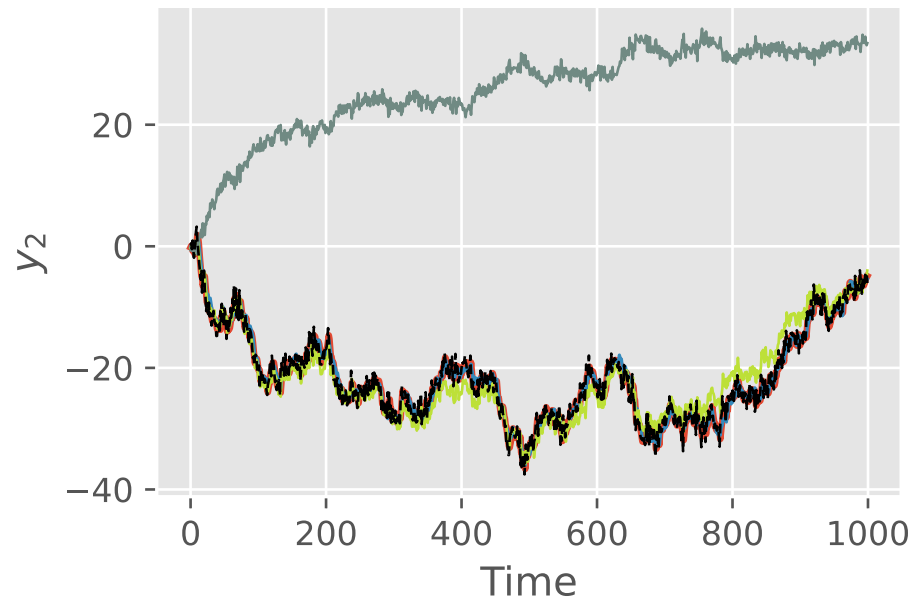

State

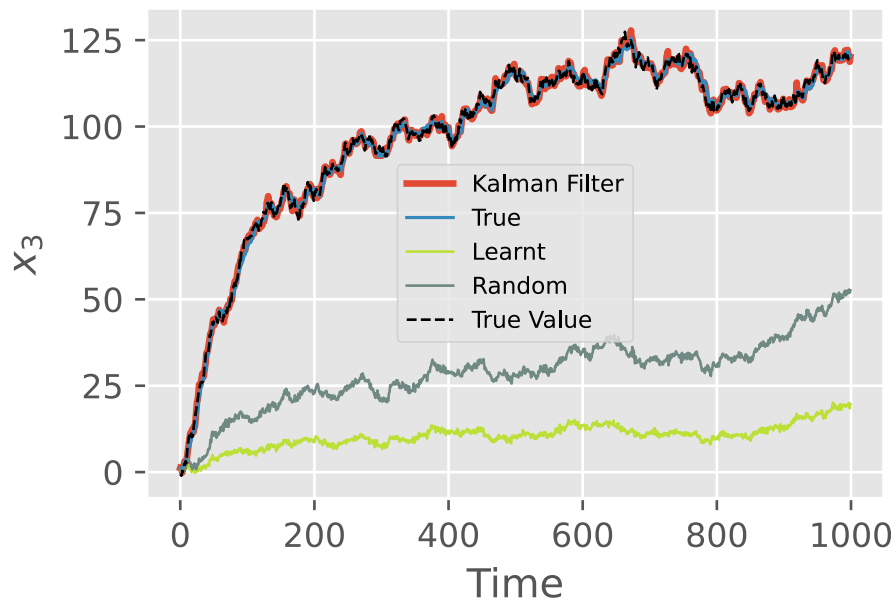

Observed

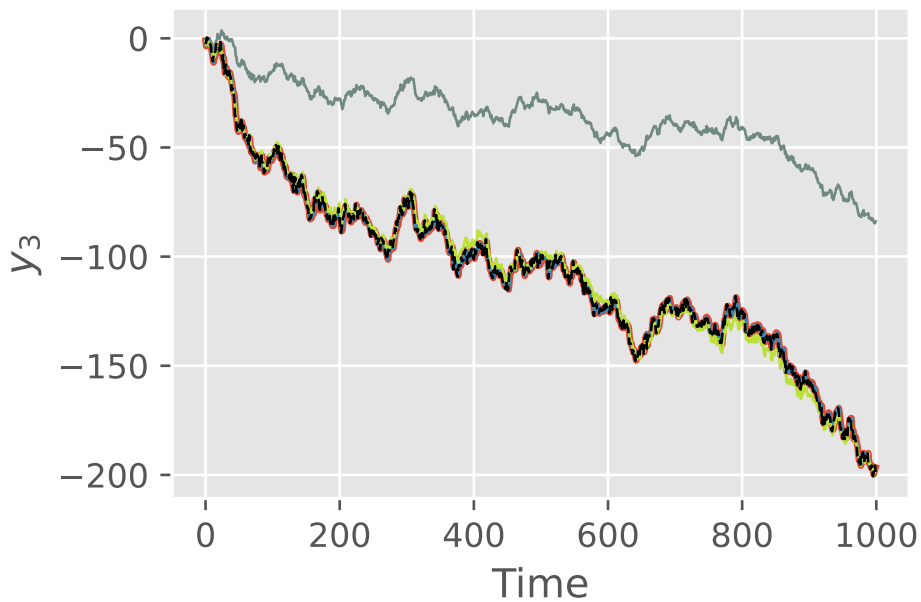

Supplement: S2 Fig — All values are with arbitrary units (a.u.). (PDF) [file pcbi.1011183.s005.pdf]
