# Supplementary material for: Predictive coding networks for temporal prediction
Source: PLoS Comput Biol. 2024 Apr 1;20(4):e1011183. doi: 10.1371/journal.pcbi.1011183 (PMC11008833; doi:10.1371/journal.pcbi.1011183)
Supplement: Attachment — Submitted filename: Review_PCOMPBIOL-D-23-00771.pdf [file pcbi.1011183.s006.pdf]

Summary:

The authors present a temporal predictive coding framework that can be implemented in simple recurrent networks, the units of which receive local inputs and the learning relies on local Hebbian plasticity. The network allows for the processing of sequences of stimuli that dynamically change over time, thereby incorporating the temporal dimension of signals. The authors show that in a linear regime, the model is a close approximation of the Kalman filter. But unlike the traditional implementations of the Kalman filter, the model can learn the parameters of the system online. Furthermore, they illustrate with a few examples that the temporal predictive coding model can also perform well on non-linear tasks. I think it would therefore be interesting to both neuroscience and ML crowd.

The paper is very well written and the maths is overall clearly presented (please see my few comments below though). While I do think that the topic is interesting, I have a few points that would need clarification (see below). Please note that not all of my comments must be answered by changing the manuscript (sometimes answering the reviewer is enough ;)).

More general comments/points:

- The process and observation noise is not estimated by the system, is it? You mention that the scaling can be done through additional inhibitory connections but how is it estimated in the first place? Did I miss something (sorry if I did). Do you give it to the network directly?
- Similarly, how does the network have access to the derivative of the function f(x)? f(x) cannot be interpreted as the activation function of a neuron, can it? So, how is f(x) chosen and how does the network computes it? I am asking more to get a better understanding for the underlying biological network implementation.
- I am wondering a bit about the time scales in your network and example tasks.
  - Your network units don't have a specific time constant, do they? Or in other words the time constant is 1. What does it imply for the tasks the network can perform?
  - Your algorithm requires the units to reach a steady state (at least this is how I interpret „Then, we iterate Equation 10 until convergence for a given observation y_k.“). You also test a version in which you only iterate one step. I would assume that the performance of the one-step algorithm depends also strongly on the time scales of the underlying true generating model and the time scales in the network
  - How do „high integration time steps“ as you suggested in the linear case resonate with the time scales of neurons and network?

Figure & task/result-specific comments:

- How do the results depend on the initialisation (probably not)
- Maybe you can show the other dimensions in a supplementary material figure (instead of just saying that the other dimensions have a similar performance)?
- The examples you show are rather low-dimensional, can you show or comment on higher dimensional examples
- At the moment your networks have as many units (x) as variables of the underlying generative process. How would neurons share the task in a larger network?
- When you learn the matrices A and C your network fails to estimate the latent space due to an under-determined system. That makes sense but also means that the model of the world is wrong, is it?
- You state „Firstly, core visual functions can often occur within 100-200 ms (Carlson, Tovar, Alink, & Kriegeskorte, 2013; Keysers, Xiao, Földiák, & Perrett, 2001; Thorpe, Fize, & Marlot, 1996; Thunell & Thorpe, 2019) which is too short to allow multiple steps of recurrent optimization, thus demonstrating that some kind of rapid single-step inference is possible.“ I am wondering if one can also interpret it as the brain relies more on predictions early in the processing!?

- You state *„Such an architecture can reconcile predictive coding networks with the lack of strong evidence for there being explicit prediction error neurons in the cortex (Walsh et al., 2020), […]“.* While I do see that there is fewer evidence for sensory PE neurons, there are more and more experimental studies showing neuronal response patterns that are are consistent with the ideas of explicit PE neurons. For instance, see the work by Georg Keller. Also, even in the network architecture 2C you would make use of PE neurons, don't you?
- In your simple recurrent network, you don't have different cell types. 1) If I see that correctly, the units in your network do not abide by Dale-s law, do they? 2) Can you imagine a task in which your network would require different cell types? Just wondering … .
- Regarding your figures: Even if your variable has arbitrary units, it would be nice if you could state that (for instance with a.u.)
- In 5D, what do the colours denote? Wouldn't it be visually more pleasing to have a colorbar and get rid of the numbers/
- Isn't it rather clear that the non-linear temporal predictive coding model outperforms the linear one when tested on non-linear tasks? Maybe I missed something, sorry.

Equation-related comments:

- Please make sure to clearly state and visualise which variables are vectors. While this might be clear from the context, it would help a more tired reader. :) You could add it in your table 1 or state it in the text.
- I think you sometimes use „log“ and sometimes you use „ln“. Please make sure to use one consistently throughout the manuscript. Otherwise, people might think you refer to two different logarithms of different basis.
- With the Gaussian assumptions in place, and Eqs. 3 and 4, I think you are missing a 1/2 in front of the expression in Eq. 6. In addition to that I am not sure why you neglect several other terms coming from the denominator of the multivariate normal distribution. Probably you have a good reason for that but it would be great if you could state it more explicitly.
- If Eq. 6 is correct (see above), then I think in Eqs. (10) and (11) a 2 is missing each.
- The same would apply to the equations (34) and (35) in the appendix.
