# Supplementary material for: Predictive coding networks for temporal prediction
Source: PLoS Comput Biol. 2024 Apr 1;20(4):e1011183. doi: 10.1371/journal.pcbi.1011183 (PMC11008833; doi:10.1371/journal.pcbi.1011183)
Supplement: Attachment — Submitted filename: response.pdf [file pcbi.1011183.s007.pdf]

Responses

↪ We thank the Editor and the Reviewers for very useful feedback and suggestions. We addressed all of them, as explained in detail below, and we feel that they significantly improved the manuscript.

Reviewer #1

"...how could you map this model onto a population of neurons in V1 (or any sensory system)? could it learn to predict from spatiotemporal patterns in images? Or is there any neural system at all in the brain that you could map this model onto?"

↪ *Mapping the model to real neural populations.* Thank you very much for pointing this out. We have added an experiment where we trained the model on patches from natural movies These results are presented in Fig 6 and described in a new section "Training tPC with natural movies". The hidden neurons trained with this dataset present motion-sensitive edge detectors, mappable to neurons in the visual cortex.

"There is much made about the disconnection between two different time-scales: the index k that indexes x(k), x(k-1), etc. vs. the time over which optimization is performed. Also the problem of having to "memorize" the past state $x_k - 1$ is discussed. But is this really a problem? In any real neural system, you could have neurons evolving at different timescales. Some are lagged, some non-lagged (as you see in LGN for example). And the sensory input evolves at one timescale, while the rate at which neurons internally can change their activity could evolve at a faster timescale. For example Reinagel has shown that LGN neurons can encode with much higher temporal precision than the timescale of visual signals captured by the cones. The paper would be much more interesting from a neurobiological perspective if it could seamlessly integrate these different timescales into one model, rather than the algorithmic solution of having a "fake time" advancing k-1 to k, and another time for optimization."

↪ *Time-scale.* Thank you for pointing this out. Indeed the need to store a working memory of the past state isn't a problem. We have changed our narrative accordingly:

> "*· · · need to store and hold fixed the state estimate of the previous step while the iterative inference of the state estimate for the current step is ongoing. · · ·*" — *Page 9, Lines 289-291*

We have made the time constant of neural dynamics explicit by introducing the parameter describing it τ to all equations describing neural dynamics. We have also used a biologically realistic time constant of visual cortical neurons into our simulations with the natural receptive fields in Fig 6. The hidden neurons' time constant and the observed movies now share the same unit of time (ms). We found that with the realistic time constant controlling the dynamics of neurons, our model can still produce realistic, motion-sensitive STRFs.

"The under-determined nature of the solution discussed with respect to Figure 4 seems to point to the fact that the model is not identifiable. Part of this would stem

from the assumption of a Gaussian prior. It may thus be desirable to think about using sparse priors over the latent variables x."

> ↪ *Sparse constraint.* This is indeed a nice intuition. In our experiment with natural movies we found that a sparse constraint on the latent variables is indeed needed to produce Gabor-like receptive fields.

Reviewer #2

"Why were the precision matrices, which are arguably quite important, not empirically explored especially on the smaller scale/toy problems examined in this work? ";
"I do expect to see (or if this is done, made explicit and very clear) in the "Learning" subsection experiments how PC with and without learning its covariance/precision matrices performs"

> ↪ *Noise precision matrices.* Thank you very much for pointing this out. We performed empirical examinations into this issue and found that both the recurrent weights A and forward weights C can encode the noise precision matrices Σ_x and Σ_y. This is shown in new Fig 5 and described in an additional section "Learning the noise covariance matrices". Therefore, the tPC model can indeed learn the noise precision matrices, without representing it explicitly in its computations and circuit implementations. This is similar to what was found in Tang et al. (2023). However, due to the complexity introduced by the temporal dimension, we leave the analytical understanding of how the weights of tPC learn the precision matrices to future studies.

"Given the focus on smaller scale and synthetic tracking problems, I would also expect to see a comparison to a few other relevant estimators, such as scented/extended KF models "

> ↪ During this revision we have already extended the simulation substantially following the comments of the Reviewer, by including learning of covariance matrices (see above) and training on natural images (see below). Following a discussion with the Editor, we feel that the paper is addressed to computational biology audience and hence further comparison with these extended Kalman filters in beyond the scope of the manuscript.

"How would this approach scale to higher dimensional latent state and observation state problems?"; "This study should also include at least one (if not two) real-world time series or dynamics dataset to compare the temporal PC model"

> ↪ We have added an experiment with high-dimensional inputs from natural movies. The training data consists of 16 patches from natural movies and to accommodate the input dimension, we used a tPC model with latent dimension 320. These results are presented in Fig 6 and described in a new section "Training tPC with natural movies".

"Typos/Revisions Found (though others might have been missed):"

> ↪ We have corrected them according to the reviewer's suggestions.

Reviewer #3

"The process and observation noise is not estimated by the system, is it? "

> ↪ *Noise precision matrices.* Thank you very much for pointing this out. We performed empirical examinations into this issue and found that both the recurrent weights A and forward weights C can encode the noise precision matrices Σ_x and Σ_y. This is shown in Fig 5 and described in a new section "Learning the noise covariance matrices". Therefore, the tPC model can indeed learn the noise precision matrices, without representing it explicitly in its computations and circuit implementations. This is similar to what was found in Tang et al. (2023).

"how does the network have access to the derivative of the function f(x)? f(x) cannot be interpreted as the activation function of a neuron, can it? So, how is f(x) chosen and how does the network computes it? I am asking more to get a better understanding for the underlying biological network implementation. "

> ↪ *Nonlinearity.* In terms of the biological implementation of the nonlinear function, we have added a sentence in the main text referring the readers to previous works discussing how to implement the nonlinearity in a predictive coding network, although implementing the nonlinearity doesn't affect the implementation of tPC overall:
>
> > *"··· The nonlinear function $f(\cdot)$ can be implemented in this circuit following the way specified in [26], through local inhibitory neurons. ··· "*
> > *— Page 8, Lines 255-257*

"I am wondering a bit about the time scales in your network and example tasks. - Your network units don't have a specific time constant, do they? Or in other words the time constant is 1. What does it imply for the tasks the network can perform? - Your algorithm requires the units to reach a steady state (at least this is how I interpret Then, we iterate Equation 10 until convergence for a given observation yk.). You also test a version in which you only iterate one step. I would assume that the performance of the one-step algorithm depends also strongly on the time scales of the underlying true generating model and the time scales in the network - How do high integration time steps as you suggested in the linear case resonate with the time scales of neurons and network? "

> ↪ Thank you for pointing out the possibility of integrating a time constant into the inference dynamics. In our previous experiments all the neurons have a time constant 1. In the revised manuscript we have made the time constant of neural dynamics explicit by introducing the parameter describing it τ to all equations describing neural dynamics. We also added a new section "Training tPC with natural movies". including Fig 6 where used a constant τ based on estimates from cortical neurons and changed the number of inference iterations to match the real-world time interval of our stimuli (in ms).

"How do the results depend on the initialisation (probably not) "

↪ In our experiments we tried initializing A and C with both random Gaussian and zero matrices and did not observe substantial differences.

"Maybe you can show the other dimensions in a supplementary material figure (instead of just saying that the other dimensions have a similar performance)? "

↪ We have added a new Supplementary Figure S2 showing the performance for other dimensions.

"The examples you show are rather low-dimensional, can you show or comment on higher dimensional examples; At the moment your networks have as many units (x) as variables of the underlying generative process. How would neurons share the task in a larger network?"

↪ We addressed both questions in Fig 6 and a new section "Training tPC with natural movies", where we trained the model with higher-dimensional inputs (16-by-16 patches from natural movies) with a larger latent size not equal to input size (320)

"When you learn the matrices A and C your network fails to estimate the latent space due to an under-determined system. That makes sense but also means that the model of the world is wrong, is it? "

↪ Yes, this result suggests that the learned model is different to the data-generating model. However, the good performance on the observation level also suggests that the model can still make accurate prediction of the external world even with this different model. So at least on the **behavioral** level, the model doesn't really need to learn the correct world model. We tried to get rid of the under-determined nature of the model by adding sparse constraint to it (Fig 6) and also obtained **representations** similar to the visual system.

"You state 'Firstly, core visual functions can often occur within 100-200 ms (Carlson, Tovar, Alink, & Kriegeskorte, 2013; Keysers, Xiao, Földiák, & Perrett, 2001; Thorpe, Fize, & Marlot, 1996; Thunell & Thorpe, 2019) which is too short to allow multiple steps of recurrent optimization, thus demonstrating that some kind of rapid single-step inference is possible.' I am wondering if one can also interpret it as the brain relies more on predictions early in the processing!?"

↪ Yes, this is also a viable interpretation.

"Also, even in the network architecture 2C you would make use of PE neurons, don't you? "

↪ Recent works have shown that it is possible to even eliminate these PE neurons from the hierarchical part of PC networks. We have added this to our discussion:

> *"··· Although in Fig 2B and C we still use error neurons in the hierarchical part of the network, they can also be circumvented following the dendritic implementation in [102]. ···"* — *Page 23, Lines 853-855*

"In your simple recurrent network, you don't have different cell types. 1) If I see that correctly, the units in your network do not abide by Dale-s law, do they? 2) Can you imagine a task in which your network would require different cell types? Just wondering ..."

↪ The possibility of implementing PC with different cell types has actually been discussed in the paper 'Predictive processing: a canonical cortical computation'. We have included this citation in our discussion:

> *"··· Moreover, as was discussed in [19], it is also possible to implement tPC with differentiation between excitatory and inhibitory neurons following Dale's Law, further increasing the biological plausibility of the model. ···"* — *Page 23, Lines 855-857*

"Regarding your figures: Even if your variable has arbitrary units, it would be nice if you could state that (for instance with a.u.) "

↪ We highlighted this in our captions.

"In 5D, what do the colours denote? Wouldn't it be visually more pleasing to have a colorbar and get rid of the numbers"

↪ Thanks for the suggestion. The colors denote the strengths of weights. We have changed the numbers to a colorbar (former Figure 5 became Figure S3 in the revised manuscript).

"Isn't it rather clear that the nonlinear temporal predictive coding model outperforms the linear one when tested on nonlinear tasks? Maybe I missed something, sorry. "

↪ We agree and that's why we have moved the experiments with the nonlinear, periodic stimuli to appendix. However, we kept the experiment with pendulum in main text, because it is interesting from the perspective that the real generative process is not exactly the one assumed by tPC.

"Please make sure to clearly state and visualise which variables are vectors. While this might be clear from the context, it would help a more tired reader. :) You could add it in your table 1 or state it in the text. "

↪ We have defined the vector variables in Table 1.

"I think you sometimes use log and sometimes you use ln. Please make sure to use one consistently throughout the manuscript. Otherwise, people might think you refer to

two different logarithms of different basis. ”

↪ We have edited this, and now consistently denote logarithms by “log”.

“With the Gaussian assumptions in place, and Eqs. 3 and 4, I think you are missing a 1/2 in front of the expression in Eq. 6. In addition to that I am not sure why you neglect several other terms coming from the denominator of the multivariate normal distribution. Probably you have a good reason for that but it would be great if you could state it more explicitly. ” “If Eq. 6 is correct (see above), then I think in Eqs. (10) and (11) a 2 is missing each. The same would apply to the equations (34) and (35) in the appendix.”

↪ The denominator and other components of Gaussian density doesn’t affect our optimization so we omitted it. We have added a sentence clarifying this.

> *“· · · (we choose to omit the other terms in the multivariate Gaussian density as they do not affect the optimization over x_k and A, B, C): · · · ”*
> *— Page 6, Lines 165-166*

We have also added a $\frac{1}{2}$ to Eq 6 so there is no need for a 2 in Eqs 10 and 11 and Appendix.
